# Beyond Traditional Risk Factors: Inflammation, Asymmetric Dimethylarginine, and N-Terminal pro–B-Type Natriuretic Peptide Predict Cardiovascular Risk in Chronic Kidney Disease

**DOI:** 10.7759/cureus.97654

**Published:** 2025-11-24

**Authors:** Eneida Hoxha, Ledio Collaku, Somida Kuka, Saimir Seferi, Pandush Pojani, Xhesika Habilaj, Matilda Kambo, Elizana Zaimi (Petrela), Anyla Bulo, Margarita Resuli

**Affiliations:** 1 Department of Internal Medicine, Clinic of Internal Medicine and Hypertension, University Hospital Center "Mother Teresa", Tirana, ALB; 2 Department of Nephrology Dialysis Transplatation, University Hospital Center "Mother Teresa", Tirana, ALB; 3 Department of Toxicology, University Hospital Center "Mother Teresa", Tirana, ALB; 4 Department of Public Health, Faculty of Medicine, University of Medicine, Tirana, ALB; 5 Department of Laboratory Medicine, University Hospital Center "Mother Teresa", Tirana, ALB

**Keywords:** adma, cardio-renal-metabolic syndrome, cardiovascular risk, chronic inflammation, chronic kidney disease, endothelial dysfunction, il-6, nt-probnp

## Abstract

Background: Cardiovascular disease (CVD) is the leading cause of morbidity and mortality in chronic kidney disease (CKD). While traditional risk factors such as hypertension and diabetes are highly prevalent, they fail to fully explain the disproportionate cardiovascular (CV) burden. Non-traditional mechanisms, including inflammation, endothelial dysfunction, oxidative stress, and metabolic disturbances, are increasingly recognized.

Objective: The objective of this study is to evaluate the prevalence and interplay of traditional and non-traditional CV risk factors in CKD, with a focus on biomarkers (IL-6, asymmetric dimethylarginine (ADMA), homocysteine, uric acid) and echocardiographic findings, and to determine their ability to predict N-terminal pro-brain natriuretic peptide (NT-proBNP) levels.

Methods: We conducted a cross-sectional study of 100 CKD patients (stages I-V), of whom 21 (21%) were on hemodialysis. Demographic, clinical, laboratory, and echocardiographic data were collected. Biomarkers (IL-6 and homocysteine in all patients; ADMA in 52 patients) were measured. Correlation and multivariable regression analyses were used to identify predictors of NT-proBNP and echocardiographic abnormalities.

Results: The cohort (60% male, mean BMI 27.4 kg/m²) showed a high prevalence of hypertension (83%) and diabetes (57%). Non-traditional biomarkers were frequently elevated: IL-6 (mean 23.5 pg/mL), homocysteine (mean 28.8 µmol/L), ADMA (mean 189.4 ng/mL), and uric acid (mean 8.5 mg/dL). Valvular calcifications were present in 52% of patients. NT-proBNP was markedly elevated (mean 10,912 pg/mL) and showed significant correlations with IL-6 (r = 0.211, p = 0.002) and ADMA (r = 0.334, p < 0.001). In multivariable regression analysis, both IL-6 (β = 0.207, p = 0.039) and ADMA (β = 0.385, p = 0.005) independently predicted NT-proBNP, with ADMA demonstrating the strongest effect. Hyperhomocysteinemia correlated with valvular calcifications, while uric acid was associated with hypertension and reduced eGFR.

Conclusion: Both traditional and non-traditional factors contribute to the CV burden in CKD. Biomarkers of inflammation and endothelial dysfunction (IL-6, ADMA) are independent predictors of NT-proBNP and provide prognostic value beyond classical determinants. Incorporating these biomarkers into risk stratification models may improve prediction and guide innovative therapeutic strategies to reduce CV morbidity and mortality in CKD.

## Introduction

Chronic kidney disease (CKD) affects about 10% of the global population and has become a major public health challenge [1]. Cardiovascular disease (CVD) is the leading cause of morbidity and mortality in CKD, with patients far more likely to die of cardiovascular (CV) complications than to progress to end-stage kidney disease (ESKD) [2]. This disproportionate CV burden reflects the systemic nature of CKD and the interplay of multiple risk factors [3]. Traditional CV risk factors such as hypertension, diabetes mellitus, dyslipidemia, and smoking are highly prevalent among CKD patients and contribute substantially to disease progression and adverse outcomes [2,4]. Nevertheless, these factors alone do not account for the marked increase in CV events and conventional models such as the Framingham risk score [5] often underestimate risk in this population [6]. In fact, the European Renal Association recently highlighted that CKD represents a CV risk equivalent to diabetes mellitus and coronary artery disease, warranting its integration into routine risk assessment models [7]. Consequently, growing attention has been directed toward non-traditional mechanisms. CKD is now recognized as a systemic pro-inflammatory and pro-calcific state in which oxidative stress, endothelial dysfunction, disturbances in mineral metabolism, anemia, and the accumulation of uremic toxins act in concert to accelerate CV injury.

Among emerging biomarkers, interleukin-6 (IL-6 pg/mL), a master regulator of inflammation, has consistently been associated with left ventricular hypertrophy, progression of heart failure, and increased mortality [8]. Asymmetric dimethylarginine (ADMA, ng/mL), an endogenous inhibitor of nitric oxide synthase, represents a robust marker of endothelial dysfunction, linked to arterial stiffness, N-terminal pro-brain natriuretic peptide (NT-proBNP) elevation, and CVr mortality in both dialysis and non-dialysis populations [9]. In parallel, NT-proBNP (pg/mL) is widely used as a marker of cardiac load and remodeling, but its interpretation in CKD remains complex due to impaired renal clearance [10,11]. Understanding the interplay of these biomarkers may provide novel insights into CV risk stratification in CKD. However, there is limited evidence on the predictive role of IL-6 and ADMA in NT-proBNP elevation among CKD patients, which this study aims to address.

Hyperhomocysteinemia (µmol/L) contributes to vascular and valvular calcification through oxidative stress and endothelial damage, while uric acid (mg/dL), once considered a bystander, is increasingly recognized as a contributor to oxidative stress, hypertension, and vascular remodeling. Cardiac biomarkers also require careful interpretation in CKD. Elevated NT-proBNP and high-sensitivity troponins reflect not only impaired renal clearance but also structural cardiac stress, demanding CKD-specific thresholds [12]. Moreover, vascular and valvular calcification, observed frequently in CKD, is now understood as an active, cell-mediated process driven by inflammation and metabolic disturbances rather than passive calcium deposition [13].

Despite increasing recognition of the role of inflammation and endothelial dysfunction in CKD, evidence on the combined impact of IL-6, ADMA, and related biomarkers on cardiac remodeling remains limited. Previous studies have often examined these pathways in isolation, with heterogeneous populations and inconsistent findings. The prognostic role of homocysteine and hemoglobin is also debated, while the clinical utility of NT-proBNP in CKD continues to be questioned. These uncertainties highlight the need for integrated analyses that combine biochemical, hematologic, and echocardiographic data to better capture the multifactorial nature of CV risk in CKD [14].

Therefore, the aim of the present study was to investigate the interplay between traditional and non-traditional CV risk factors in a well-characterized cohort of CKD patients, focusing on the associations of IL-6, ADMA, homocysteine, hemoglobin, and uric acid with NT-proBNP and echocardiographic parameters. By elucidating these relationships, we sought to provide new insights into CV risk stratification in CKD and to inform future prevention strategies.

## Materials and methods

Study design and population

We conducted a cross-sectional observational study including 100 consecutive patients with CKD stages I-V, evaluated at the Department of Internal Medicine and Nephrology/Dialysis of our institution between 2023 and 2024. Patients with acute kidney injury, active systemic infection, recent myocardial infarction (≤ three months), pre-existing or decompensated heart failure, active malignancy, or autoimmune disease under immunosuppressive therapy were excluded. Inclusion criteria comprised adults (≥18 years) with a confirmed diagnosis of CKD stages I-V, stable clinical condition, and ability to provide informed consent. Of the study population, 21 patients (21%) were on chronic hemodialysis, receiving regular thrice-weekly sessions with a standard Kt/V > 1.2 (a measure of dialysis adequacy), indicating adequate dialysis efficiency. Dialysis adequacy and volume status were routinely monitored to ensure adequate clearance (Kt/V > 1.2). Blood samples for biomarker analyses were collected immediately before the mid-week dialysis session to minimize fluctuations related to fluid shifts or solute clearance. For these patients, estimated glomerular filtration rate (eGFR) was not used for staging or analysis, as it no longer provides a reliable measure of kidney function once renal replacement therapy has been initiated. Instead, these patients were classified as CKD stage V (hemodialysis) based on clinical status and treatment modality. All participants provided written informed consent. The study protocol was approved by the Institutional Ethics Committee of the University of Medicine, Tirana (UMT) (Approval No. 3285/6; November 02, 2023) and complied with the principles of the Declaration of Helsinki.

Clinical and demographic data

Demographic characteristics included age, sex, and body mass index (BMI). Clinical data were collected through structured interviews and medical record review, including comorbidities (hypertension, diabetes mellitus, dyslipidemia, smoking history, and CV disease). Blood pressure was measured in the seated position after five minutes of rest, using the average of two readings obtained with a validated device. Medication use was documented, focusing on renin-angiotensin-aldosterone system (RAAS) inhibitors, statins, beta-blockers, sodium-glucose co-transporter-2 inhibitors, non-steroidal mineralocorticoid receptor antagonist, and diuretics.

Laboratory measurements

Blood samples were collected after overnight fasting. Routine biochemical analyses included serum urea (mg/dL), creatinine (mg/dL), estimated glomerular filtration rate (eGFR mL/min/1.73 m², CKD-EPI equation [15]), hemoglobin (g/dL), fasting glucose (mg/dL), lipid profile (mg/dL), uric acid (mg/dL), calcium (mg/dL), phosphate (mg/dL), and electrolytes (sodium, potassium, chloride in mmol/L). In addition, specific biomarkers were assessed: interleukin-6 (IL-6 pg/mL) as an inflammatory marker, plasma homocysteine (µmol/L) as a metabolic marker, and NT-proBNP (pg/mL) as a cardiac biomarker, which were measured in all 100 patients, while asymmetric dimethylarginine (ADMA ng/mL), as an indicator of endothelial dysfunction, was determined in a subgroup of 52 patients. All measurements were performed in the central laboratory of our hospital and a certified external laboratory, according to standard procedures and quality control requirements.

Echocardiographic assessment

Transthoracic echocardiography was performed in all 100 patients by a single experienced cardiologist who was blinded to laboratory results, using a standardized protocol and the same commercially available ultrasound system to ensure maximal consistency and reproducibility. For patients on maintenance hemodialysis, echocardiographic evaluations were performed on non-dialysis days, at least 12-24 hours after the last session, to minimize the hemodynamic effects of fluid overload. Measurements included left ventricular mass index (LVMI g/m²), interventricular septal thickness (mm), posterior wall thickness (mm), left atrial diameter (mm), and left ventricular ejection fraction (LVEF %) (by Simpson’s biplane method [16]). Diastolic function was evaluated using Doppler transmitral flow and tissue Doppler imaging. The presence of valvular calcifications, including aortic and mitral, was systematically recorded.

Statistical analysis

Continuous variables were expressed as mean ± standard deviation (SD) or median with interquartile range (IQR), according to data distribution. Categorical variables were reported as frequencies and percentages. Group comparisons were performed using Student’s t-test or Mann-Whitney U test for continuous variables, and chi-square or Fisher’s exact test for categorical variables. Correlations between biomarkers and echocardiographic parameters were assessed with Pearson or Spearman coefficients, as appropriate. To identify independent predictors of NT-proBNP, we applied multivariable linear regression adjusting for age, sex, BMI, eGFR, and traditional CV risk factors. Multicollinearity was excluded, as variance inflation factors were <2. Statistical significance was defined as a two-tailed p-value <0.05. All analyses were performed with IBM SPSS Statistics for Windows, Version 27 (Released 2020; IBM Corp., Armonk, New York, United States).

## Results

Baseline characteristics

The study included 100 patients with CKD, of whom 60% were male. The mean age was 64.3 ± 12.1 years, and the mean BMI was 27.4 ± 5.3 kg/m². Most patients were in intermediate to advanced stages of CKD. The stage distribution according to the CKD-EPI equation [15] was as follows: stage II - 6%, stage III - 34%, stage IV - 32%, stage V (non-dialysis) - 7%, and stage V (on maintenance hemodialysis) - 21%. Hypertension was present in 83%, diabetes mellitus in 57%, and dyslipidemia in 42%. The mean eGFR was 27.5 ± 18.0 mL/min/1.73m², and mean hemoglobin concentration was 10.9 ± 2.1 g/dL.

Biomarker profiles

Non-traditional biomarkers were frequently elevated in the study population. IL-6, homocysteine (µmol/L), and uric acid (mg/dL) levels were substantially higher than reference values, while NT-proBNP (pg/mL) was markedly elevated across the cohort, reflecting significant cardiac stress. Of note, IL-6 values showed wide variability (mean 23.5 ± 27.3 pg/mL), consistent with a skewed distribution of inflammatory markers in CKD. ADMA, measured in a subgroup of 52 patients, was also increased (mean 189.4 ± 44.5 ng/mL), indicating endothelial dysfunction (Table 1).

**Table 1:** Biomarker profiles in patients with chronic kidney disease Biomarker levels in the study population compared with standard reference ranges. Values are presented as mean ± standard deviation. Reference ranges are based on commonly accepted laboratory cut-offs. Slight differences in the number of patients reflect missing data for individual biomarkers. Elevated concentrations of IL-6 (pg/mL), homocysteine (µmol/L), ADMA (ng/mL), uric acid (mg/dL), and NT-proBNP (pg/mL) reflect systemic inflammation, endothelial dysfunction, metabolic abnormalities, and cardiac strain in patients with chronic kidney disease. IL-6: Interleukin-6; ADMA: asymmetric dimethylarginine; NT-proBNP: N-terminal pro–B-type natriuretic peptide; SD: standard deviation; CKD: chronic kidney disease.

Biomarker	N (patients)	Mean+SD	Reference range
IL-6 (pg/mL)	100	23.5 ± 27.3	<7
Homocysteine (µmol/L)	100	28.8 ± 10.6	6-15
Uric acid (mg/dL)	98	8.5 ± 3.0	<7 (men), <6 (women)
ADMA (ng/mL)	52	189.4 ± 44.6	50–150
NT-proBNP (pg/mL)	99	10,912 ± 16,161	<450

Echocardiographic findings

Echocardiographic evaluation revealed structural and functional cardiac abnormalities in a large proportion of patients. Left ventricular hypertrophy (LVH, LVMI >115 g/m² in men, >95 g/m² in women) was identified in 68% and left atrial enlargement (diameter >40 mm) in 54%. Systolic dysfunction (LVEF <50%) was observed in 28%, while diastolic dysfunction was present in 63%. Valvular calcifications, either aortic or mitral, were detected in 52% of patients.

Correlation analyses

**Figure 1:**
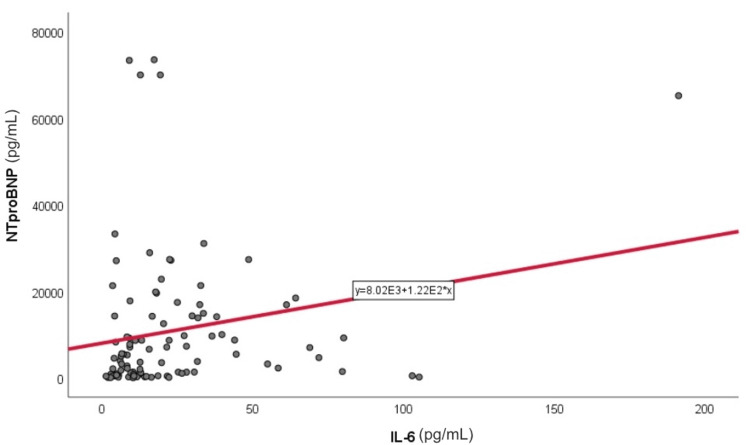
Correlation between IL-6 (pg/mL) and NT-proBNP (pg/mL) levels in patients with CKD Scatter plot showing the relationship between IL-6 and NT-proBNP levels in patients with chronic kidney disease. The solid line represents the Pearson correlation. NT-proBNP: N-terminal pro–B-type natriuretic peptide; IL-6: interleukin-6; CKD: chronic kidney disease.

**Figure 2:**
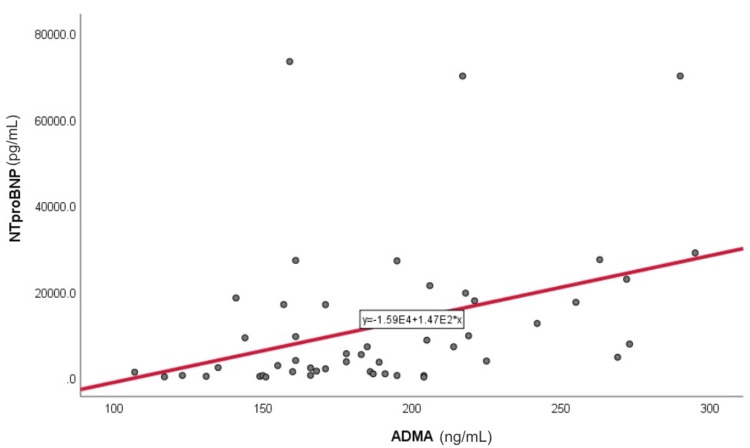
Correlation between ADMA (ng/mL) and NT-proBNP (pg/mL) levels in a subgroup of 52 patients Legend: Scatter plot showing the relationship between ADMA and NT-proBNP levels in a subgroup of 52 patients. The solid line represents the Pearson correlation. ADMA: asymmetric dimethylarginine; NT-proBNP: N-terminal pro–B-type natriuretic peptide.

Correlation analyses demonstrated significant associations between NT-proBNP (pg/mL) and both IL-6 (pg/mL) (r = 0.211, p = 0.002) (Figure 1) and ADMA (ng/mL) (r = 0.334, p < 0.001) (Figure 2). Homocysteine (µmol/L) was significantly associated with the presence of valvular calcifications (p < 0.05), whereas uric acid (mg/dL) correlated with hypertension and lower eGFR (mL/min/1.73 m²) values (p < 0.05). No significant correlations were observed between NT-proBNP and hemoglobin (g/dL) or lipid profile parameters (mg/dL) (Table 2). 

**Table 2:** Correlations between NT-proBNP levels and clinical, laboratory, and echocardiographic parameters in CKD patients Pearson correlation coefficients between NT-proBNP (pg/mL) and selected clinical, laboratory, and echocardiographic variables in patients with chronic kidney disease. Values represent Pearson correlation coefficients (r) with corresponding p-values. Differences in sample size reflect missing data for individual variables. A p-value <0.05 was considered statistically significant. CKD: Chronic kidney disease; eGFR: estimated glomerular filtration rate; IL-6: interleukin-6; ADMA: asymmetric dimethylarginine; RBC: red blood cells; LVEF: left ventricular ejection fraction; NT-proBNP: N-terminal pro–B-type natriuretic peptide.

Variable	r	p-value	n
eGFR (mL/min/1.73 m²)	0.132	0.054	100
IL-6 (pg/mL)	0.211	0.002	100
ADMA (ng/mL)	0.334	<0.001	52
Homocysteine (µmol/L)	0.014	0.868	100
Hemoglobin (g/dL)	-0.098	0.153	100
Hematocrit (%)	-0.079	0.247	100
RBC (×10⁶/µL)	-0.044	0.520	100
LVEF (%)	-0.085	0.222	100
Left atrial diameter (mm)	0.081	0.250	99
Interventricular septal thickness (mm)	0.069	0.319	100
Posterior wall thickness (mm)	-0.024	0.732	100

Multivariable regression

**Figure 3:**
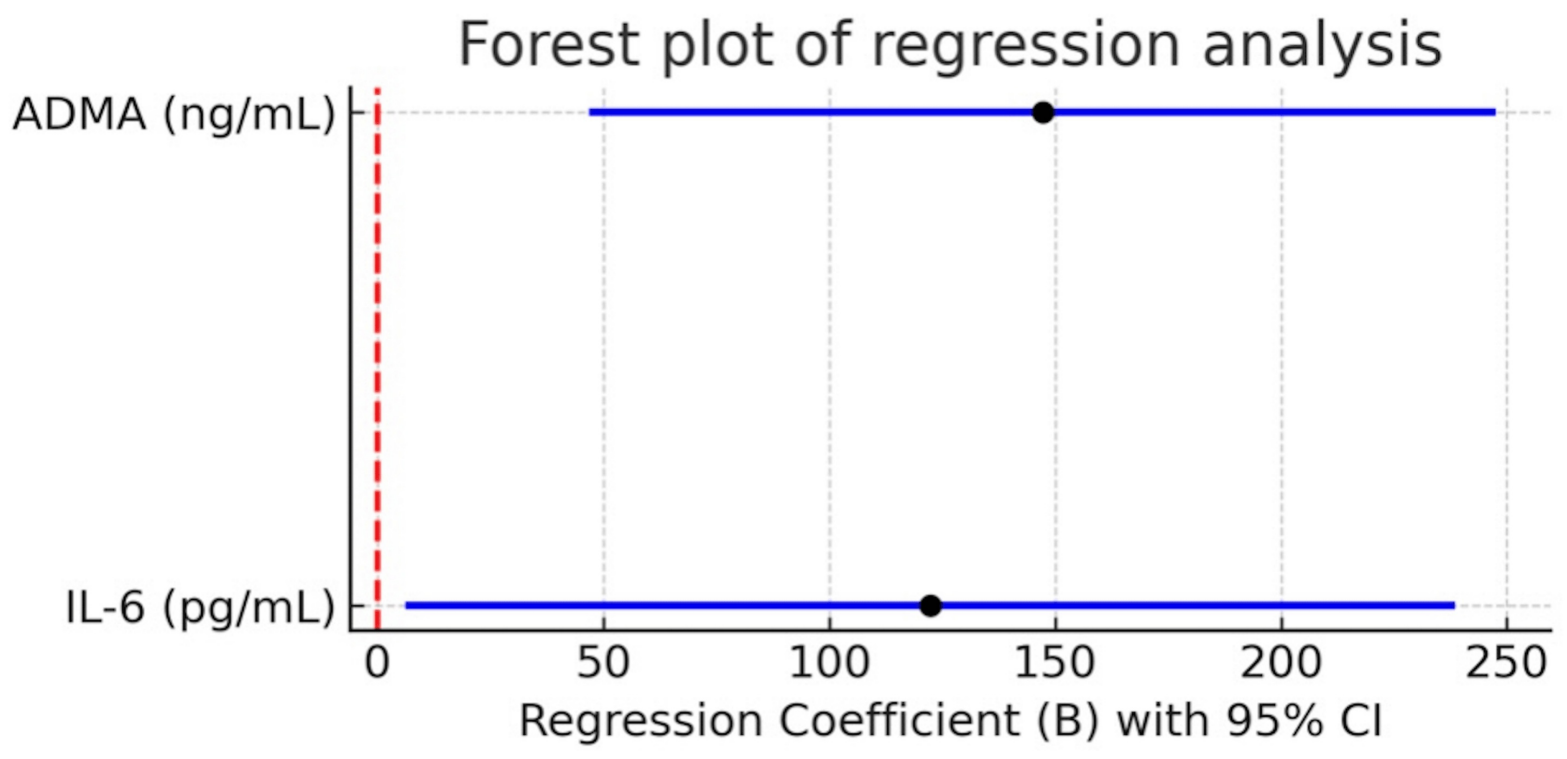
Forest plot of regression analysis for predictors of NT-proBNP (pg/ml) levels in CKD patients Forest plot displaying the results of multivariable regression analysis for predictors of NT-proBNP levels in patients with chronic kidney disease. Dots represent unstandardized regression coefficients (B) with 95% confidence intervals (CI). The vertical dashed red line indicates the null value (B = 0). IL-6: interleukin-6; ADMA: asymmetric dimethylarginine; NT-proBNP: N-terminal pro–B-type natriuretic peptide; CKD: chronic kidney disease; CI: confidence interval.

In the multivariable linear regression model adjusted for age, sex, BMI, eGFR, and traditional cardiovascular risk factors, both IL-6 (β = 0.207, p = 0.039) and ADMA (β = 0.385, p = 0.005) were identified as independent predictors of NT-proBNP, with ADMA demonstrating the strongest predictive effect (Figure 3). By contrast, neither homocysteine nor hemoglobin retained independent associations with NT-proBNP after adjustment (Table 3).

**Table 3:** Multivariable linear regression analysis of predictors of NT-proBNP in CKD patients Multivariable linear regression model examining IL-6 (pg/mL) and ADMA (pg/mL) as independent predictors of NT-proBNP (pg/mL) levels in patients with chronic kidney disease (n = 100; ADMA available in 52 patients). Values are expressed as unstandardized regression coefficients (B) with standard errors (SE), standardized coefficients (β), and 95% confidence intervals (CI). A p-value <0.05 was considered statistically significant. IL-6: interleukin-6; ADMA: asymmetric dimethylarginine; NT-proBNP: N-terminal pro–B-type natriuretic peptide; SE: standard error; CI: confidence interval; CKD: chronic kidney disease.

Variable	B (Unstandardized)	SE	β (Standardized)	95% Cl for B	p-value
IL-6 (pg/mL)	122.3	58.5	0.207	6.1-238.5	0.039
ADMA (ng/mL)	147.2	50.0	0.385	46.8-247.6	0.005

## Discussion

Main findings

In this cross-sectional study of 100 patients with CKD, we confirmed a high prevalence of traditional CV risk factors, including hypertension, diabetes, and dyslipidemia. However, these factors alone did not account for the markedly elevated NT-proBNP (pg/mL) levels observed in our cohort. Non-traditional biomarkers were frequently abnormal, with IL-6 (pg/mL), ADMA (ng/mL), and homocysteine (µmol/L) significantly elevated in the majority of patients. Both IL-6 and ADMA emerged as independent predictors of NT-proBNP, with ADMA exerting the strongest effect, while homocysteine correlated with valvular calcifications. Elevated uric acid (mg/dL) levels were also common and associated with metabolic derangements and impaired kidney function. Together, these findings highlight the multifactorial nature of CV burden in CKD, where inflammation, endothelial dysfunction, and metabolic abnormalities converge to accelerate myocardial and vascular injury [17]. Although NT-proBNP and ADMA levels appeared higher in patients with advanced CKD (stages IV-V), these differences did not reach statistical significance, possibly reflecting limited sample size and stage heterogeneity.

Relation to literature

Our results are in line with previous evidence showing that conventional models such as the Framingham risk score tend to underestimate CV risk in CKD populations. Recent position statements from ERA and KDIGO emphasize that CKD should be regarded not only as a renal condition but also as a systemic, pro-inflammatory, and pro-calcific state and a core component of the emerging cardio-kidney-metabolic (CKM) syndrome [3,18].

Chronic inflammation has long been recognized as a key driver of CV remodeling. In our cohort, elevated IL-6 levels were associated with NT-proBNP and left ventricular hypertrophy, in agreement with prior studies linking IL-6 to myocardial fibrosis, heart failure progression, and mortality [19,20]. IL-6 promotes endothelial activation, vascular calcification, and adverse cardiac remodeling, underscoring its dual role as a biomarker and a potential therapeutic target [19,21].

Endothelial dysfunction, reflected by ADMA, was another critical determinant. ADMA is an endogenous inhibitor of nitric oxide synthase that reduces nitric oxide bioavailability, thereby promoting oxidative stress, vasoconstriction, and arterial stiffness [22]. In our cohort, ADMA showed the strongest independent association with NT-proBNP, suggesting a direct link between endothelial dysfunction and subclinical cardiac stress. This observation is consistent with previous studies reporting that elevated ADMA predicts CKD progression, CV events, and mortality in both dialysis and pre-dialysis patients [23], which reinforces its potential utility as a prognostic biomarker in clinical practice.

Hyperhomocysteinemia, which was highly prevalent in our study, showed a significant association with valvular calcifications. This finding supports earlier evidence that homocysteine promotes oxidative stress, endothelial injury, and vascular smooth muscle proliferation, thereby accelerating vascular and valvular mineralization [24]. Valvular calcification is increasingly recognized as a marker of systemic vascular disease in CKD, with important prognostic implications for morbidity and mortality [25].

Finally, elevated uric acid levels correlated with hypertension and lower eGFR (mL/min/1.73 m²) in our patients. Although its causal role remains debated, growing evidence suggests that uric acid actively contributes to vascular remodeling and oxidative stress rather than serving merely as a passive marker, consistent with findings from recent meta-analyses [26,27].

Multivariable regression and novel insights

In multivariable regression analysis, both IL-6 and ADMA remained independent predictors of NT-proBNP, whereas hemoglobin and homocysteine did not retain statistical significance. Notably, NT-proBNP showed no significant associations with either hemoglobin or lipid profile parameters, in contrast to some previous reports. Several factors may account for these discrepancies. First, the predominant influence of non-traditional risk factors, particularly inflammation and endothelial dysfunction, in our cohort may have overshadowed weaker associations with anemia and dyslipidemia. Second, the heterogeneity of CKD stages, including the presence of both pre-dialysis and dialysis patients, likely introduced variability in hemodynamic and metabolic profiles that diluted such relationships. Third, the impact of therapeutic interventions, such as erythropoiesis-stimulating agents, iron supplementation, and statins, may have attenuated the potential impact of hemoglobin and lipid levels on cardiac biomarkers. Finally, the markedly elevated NT-proBNP values observed in our population are likely to reflect advanced myocardial remodeling and hemodynamic overload [28], processes that are less directly influenced by hemoglobin concentration or lipid metabolism.

Clinical implications

These findings carry important clinical implications. While traditional risk factors remain relevant, they are insufficient to capture the full spectrum of CV risk in CKD. Biomarkers of inflammation (IL-6) and endothelial dysfunction (ADMA) provide complementary prognostic information and could be considered for integration into future risk stratification models [29]. Homocysteine and uric acid may also serve as useful markers to identify patients at higher risk for vascular and valvular calcification. Importantly, elevated NT-proBNP in CKD should not be interpreted solely as a consequence of impaired renal clearance but rather as a reflection of pathophysiological stressors, including inflammation and endothelial dysfunction [10,30]. From a therapeutic standpoint, interventions targeting inflammatory pathways (such as the IL-6 axis) and strategies aimed at restoring nitric oxide bioavailability represent promising avenues. Likewise, approaches to reduce oxidative stress, homocysteine, and uric acid levels may contribute to mitigating vascular and valvular complications. Overall, a multidimensional approach that addresses both traditional and non-traditional risk factors has the potential to improve CV outcomes, survival, and quality of life in patients with CKD.

Limitations

This study has several limitations that should be acknowledged. First, its cross-sectional design precludes any causal inference between biomarker levels and CV outcomes. Second, the overall sample size was modest and particularly limited for ADMA (n = 52), which may have reduced statistical power to detect weaker associations. Biomarker measurements were performed at a single time point and may not reflect longitudinal variability. Third, subgroup analyses stratified by CKD stage and dialysis status were constrained by small numbers, limiting the robustness of multivariable modeling. In addition, the cohort was recruited from the Department of Internal Medicine and Nephrology/Dialysis of a single center, which may restrict the generalizability of our findings to broader CKD populations. Finally, the absence of longitudinal follow-up prevented assessment of the prognostic value of biomarkers for incident CV events. Additionally, despite all dialysis patients achieving adequate Kt/V targets, minor variations in dialysis efficiency could still have influenced biomarker concentrations; this was acknowledged as a potential confounding factor.

Despite these limitations, the study provides valuable contributions by integrating clinical, laboratory, and echocardiographic data and by highlighting the contribution of inflammation, endothelial dysfunction, and metabolic abnormalities to cardiac stress in CKD. Future large, multicenter, prospective studies are warranted to validate these findings and to determine their utility for CV risk prediction in CKD.

Perspectives

Despite these limitations, our findings provide valuable insights into the interplay between traditional and non-traditional risk factors in CKD. Future research should focus on validating these results in larger, multicenter, prospective cohorts and on evaluating the prognostic significance of IL-6, ADMA, and homocysteine in long-term follow-up. Interventional studies targeting inflammatory pathways, nitric oxide bioavailability, and oxidative stress are urgently needed to clarify the causal mechanism and explore the therapeutic potential. Furthermore, the integration of non-traditional biomarkers into CV risk prediction models for patients with CKD may enhance precision medicine, allowing improved stratification and individualized management strategies to reduce the disproportionate CV burden in this vulnerable population.

## Conclusions

In this study of 100 patients with CKD, we showed that CV risk is shaped not only by traditional factors but also by non-traditional determinants such as inflammation, endothelial dysfunction, hyperhomocysteinemia, and uric acid (mg/dL) abnormalities. Both IL-6 (pg/mL) and ADMA (ng/mL) emerged as independent predictors of NT-proBNP (pg/mL), with ADMA exerting the strongest effect, while homocysteine (µmol/L) was associated with valvular calcifications. These findings confirm that CKD is a systemic condition in which chronic inflammation, endothelial dysfunction, and metabolic derangements converge to accelerate cardiovascular injury.

Our results highlight the importance of integrating non-traditional biomarkers into cardiovascular risk stratification in CKD. Incorporating IL-6 and ADMA into clinical risk models may help refine prediction beyond traditional factors, whereas homocysteine and uric acid could help identify patients at risk for vascular and valvular calcification. Clinically, NT-proBNP should be recognized not only as a marker of impaired clearance but also as an indicator of advanced cardiac remodeling and hemodynamic stress. Future multicenter and longitudinal studies are warranted to validate these findings and to determine whether targeting inflammation, nitric oxide bioavailability, and oxidative stress can translate into improved outcomes for this high-risk population.
